# Perspectives on Endogenous Opioids in Birds

**DOI:** 10.3389/fphys.2018.01842

**Published:** 2018-12-21

**Authors:** Colin G. Scanes, Krystyna Pierzchala-Koziec

**Affiliations:** ^1^Center of Excellence in Poultry Science, University of Arkansas, Fayetteville, AR, United States; ^2^Department of Animal Physiology and Endocrinology, University of Agriculture in Krakow, Kraków, Poland

**Keywords:** β endorphin, Met-enkephalin, endogenous opioids, opioid receptor, stress

## Abstract

The present review summarizes the state of knowledge of endogenous opioids in birds. Endogenous opioid peptides acts in a neuromodulatory, hormonal and paracrine manner to mediate analgesic and other physiological functions. These peptides act through specific G-protein coupled receptors. Opioid receptors consist of a family of four closely-related proteins. The three types of opioid receptors are the mu (MOR or μ), delta (DOR or δ), and kappa (KOR or κ) opioid receptor proteins. The role of the fourth member of the opioid receptor family, the nociceptin or orphanin FQ receptor (ORL), is not clear. The ligands for opioid receptors are: β –endorphin (MOR), Met- enkephalin, Leu-enkephalin (DOR) and dynorphin (KOR), together with probably endomorphins 1 and 2. In spite of long history of research on endogenous opioid peptides, there are no studies of endogenous opioids *per se* in wild birds and few in poultry species. β-endorphin is present in all birds investigated and there is close agreement between the structures of β-endorphin in different birds. Plasma concentrations of β-endorphin are increased by ether stress in geese. There is evidence that β-endorphin plays a role in the control of luteinizing hormone release in chickens. Met-enkephalin is present in tissues such as the retina, hypothalamus, pituitary gland, and adrenals together with circulation of birds. Stresses such as crowding and withholding water increase circulating concentrations of Met-enkephalin in chickens. The structures of chicken dynorphin A and B have been deduced from cDNA. What is missing are comprehensive studies of plasma concentrations and expression of the full array of endogenous opioids in multiple avian species under different situations. Also, what is not known is the extent to which circulating or locally released or intra-cellular Met-enkephalin influence physiological process in birds. Thus, there is considerable scope for investigation of the physiology of endogenous opioids in birds.

## Introduction

The ligands for opioid receptors are endogenous opioid peptides, specifically: Met- enkephalin, Leu-enkephalin, β -endorphin and dynorphin, together with probably endomorphins 1 and 2. These are the physiological signaling peptides that mimic the effects of synthetic and natural opioids such as morphine and codeine.

**Figure 1:**
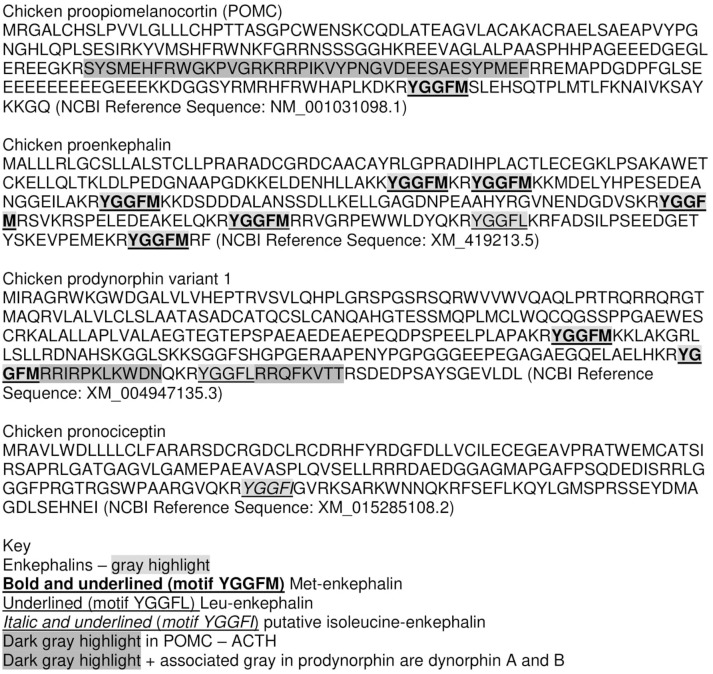
Structures of avian endogenous opioids deduced from cDNA.

The opioid/orphanin gene family contains the following:

- Proenkephalin A (PENK) gene—encoding pro-enkephalin which is processed to the endogenous opioids—Met-enkephalin and Leu-enkephalin (Figure 1).
- Proopiomelanocortin (POMC) gene—encoding POMC which is processed to the endogenous opioid–β-endorphin (containing the amino-acid sequence of Met-enkephalin) together with adrenocorticotropin (ACTH) and other peptides (Figure 1).
- Prodynorphin (proenkephalin B, PDYN) gene–encoding pro-dynorphin which is processed to the endogenous opioid–dynorphin (Figure 1).
- Pronociceptin/proorphanin FQ (PNOC) gene–encoding pro-prepronociceptin which is processed to a series of peptides including nociceptin, nocistatin, and orphanin FQ2 (Figure 1).

The opioid receptors are members of the G-protein coupled receptors (GPCR) gene family and the rhodopsin-like superfamily of GPCR (reviewed Stevens, 2009):

- Delta (δ) opioid peptide receptor (DOR or DOPr or OP_1_ receptors) (d as in vas deferens). Endogenous ligand: Met enkephalin and Leu enkephalin together with probably β-endorphin.
- Mu (μ) opioid peptide receptor (MOR or MOPr or OP_3_ receptors) (m as in morphine). Endogenous ligand: β-endorphin and probably enkephalins and endomorphins 1 and 2.
- Kappa (κ) opioid peptide receptor (KOR or KOPr or OP_3_ receptors) (k as ketocyclazocine). Endogenous ligand: dynorphin and possibly other.
- Nociceptin or orphanin FQ receptor (ORL) or opioid receptor-like receptor OPRL1. Endogenous ligand: the neuropeptide, nociceptin or orphanin FQ (Grossman and Clement-Jones, 1983; Mansour et al., 1995; reviewed Stevens, 2009).

These endogenous opioid receptor agonists have analgesic (relieving pain) or antinociceptive (inhibiting the sensation of pain) properties. There are other peptides that bind to opioid receptors. For instance, the tetrapeptide, cytochrophin-4, is a breakdown product of cytochrome-b and influences memory formation in chicks (Freeman and Young, 2000). The carboxy-amidated tetrapeptides, endomorphins 1 and 2, have been isolated from bovine brain tissue (Zadina et al., 1999). However, the endomorphins have received little attention in avian species.

**Figure 2:**
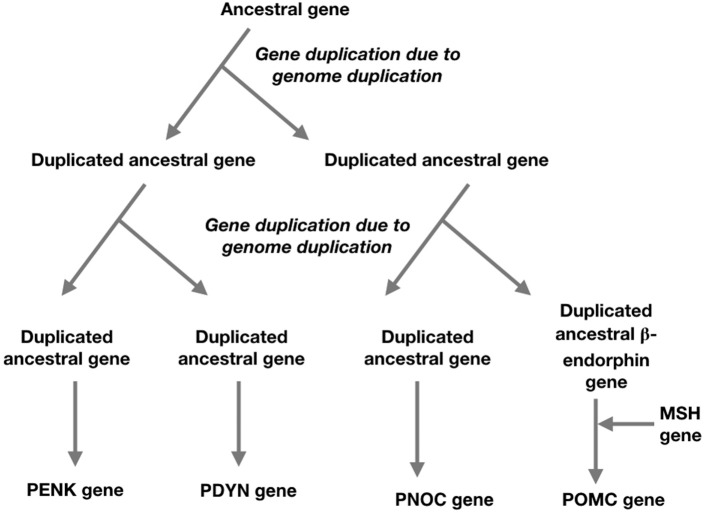
A schema for the evolution of the opioid/orphanin gene family based on two genome duplications and the insertion of melanocortin sequence to ancestral beta-endorphin gene (based on Sundström et al., 2010; Navarro et al., 2016).

It is thought that the four opioid receptor genes and the four members of the opioid/orphanin family of genes are the result of two genome duplications leading to quadruplication of the ancestral genes; these occurring prior to the radiation of the Gnathostomes (Figure 2) (Khalap et al., 2005; Dreborg et al., 2008; Sundström et al., 2010). It has not been possible to identify pro-enkephalin genes in primitive vertebrates (Agnathans) or basal chordate (Dreborg et al., 2008; Sundström et al., 2010).

The present review summarizes the state of knowledge of endogenous opioids in birds. In addition, areas ripe for research are covered. The communication discusses endogenous opioids in the following order: β-endorphin, Met-enkephalin and finally, products of the prodynorphin.

## β-Endorphin in Birds

There is arguably more information on β-endorphin than of any other endogenous opioid in birds. This is particularly the case with the deduced structures of POMC from multiple species of birds together with mammals and reptiles.

### Evolutionary Aspects of β-Endorphin

**Figure 3:**
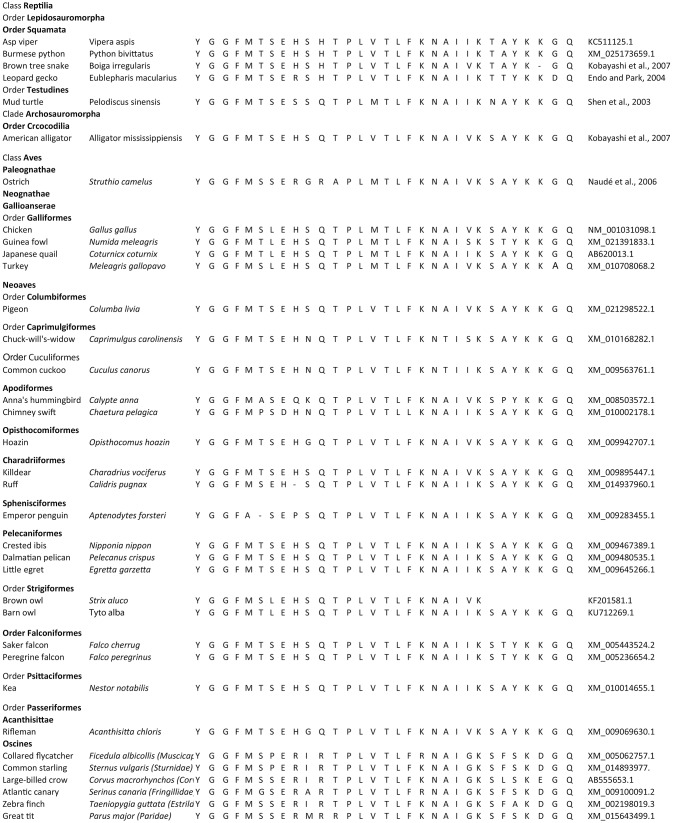
Evolution of β-endorphin. Comparison for the structures of β-endorphin in reptiles and birds.

**Figure 4:**
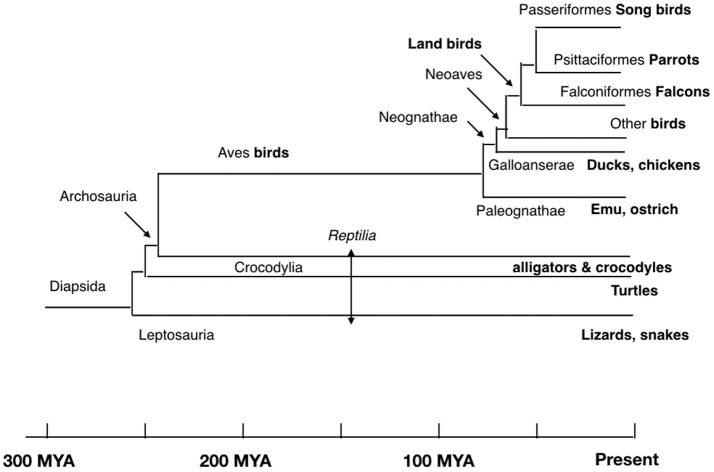
Evolutionary relationships of birds and reptiles showing conjectural time line (based on Hedges and Kumar, 2009; Chiari et al., 2012; Ezcurra et al., 2014).

β-endorphin contains the Met-enkephalin pentapeptide motif (Figure 1). The deduced structures of β-endorphin in reptiles and birds together with evolutionary relationships are summarized in Figures 3, 4 (Shen et al., 2003). Despite the last common ancestor of reptiles and birds being about 250 million years ago, there is very close similarities between the structures of β-endorphin in reptiles and birds (Naudé et al., 2006; Shoureshi et al., 2007; Dores and Baron, 2011). A tentative structure of ancestral avian β-endorphin in the common ancestor of both birds and *Crocodilia* (i.e., within the Archosauromorpha, the clade includes birds, crocodyles and dinosaurs) is the following:

YGGFMXSEHSQTPLVTLFKNAIVKSAYKKGQ (Where X is S or T) (Endo and Park, 2004).

This is identical to β-endorphin in both alligator and pigeon (Kobayashi et al., 2007). Within the class *Aves*, there are close similarities for β-endorphin. Excluding residue 6, there are two or fewer amino-acid residue differences between the ancestral form and that in multiple birds including chicken, egret, ibis, pelican, rifleman, and ruff (Figures 3, 4). This argues strongly that there is strong evolutionary pressure to maintain the structure of β-endorphin and, therefore, also of the physiological importance of β-endorphin in birds. In contrast, there are multiple differences in the *Oscines* (song birds) (Figures 3, 4).

### Circulating Concentrations of β-Endorphin

There is limited information on circulating concentrations of β-endorphin in either wild or domesticated birds. In plasma from adult chickens, two-thirds of β-endorphin immune-reactivity measured by radioimmunoassay followed by Sephadex G-75 chromatography has an identical size as β-endorphin but one-third of β-endorphin is the same size as β-lipotropin (Hylka and Thommes, 1991).

The later research on domestic gander proved that plasma concentrations of immunoreactive β-endorphin measured by specific radioimmunoassay are about 30 pmoles · ml^−1^ (Barna et al., 1998a).

It might be assumed that circulating concentrations of β-endorphin would change in parallel with those of ACTH as they are both products of POMC. However, this does not seem to be necessarily the case. While, plasma concentrations of ACTH increased following castration in domestic geese, there is no effect on circulating concentrations of β-endorphin (Barna et al., 1998a). Moreover, while plasma concentrations of ACTH in domestic geese are increased by both ether stress or LPS endotoxin, plasma concentrations of β-endorphin are reported to be elevated following ether stress but not LPS endotoxin (Barna et al., 1998b). Thus, there is some evidence of independent control of synthesis and/or release and/or degradation of β-endorphin and ACTH.

### Distribution of β-Endorphin and Tissue Concentrations

There are reports on the immunocytochemical studies of the regional distribution of β-endorphin containing neurons in avian brains (Bayon et al., 1980; Contijoch et al., 1993; Van Gils et al., 1994). β-endorphin as determined by radioimmunoassay has been reported to vary in the hypothalamus as follows: medial basal hypothalamus (MBH) (1.6 pmoles · mg^−1^) > paraventricular nucleus > pars nervosa/supra-optic nucleus > septum >> archistriatum/lobus parolfactorius (Barna et al., 1998a). Hypothalamic concentrations of β-endorphin are decreased at the time of spontaneous and progesterone induced pre-ovulatory LH surge (Contijoch et al., 1993).

β-endorphin is present in the goose anterior pituitary gland at lower concentrations than that of ACTH as follows: cephalic lobe: β-endorphin-867 pmol · lobe^−1^, ACTH-3596 pmol · lobe^−1^ (ratio 1:4.15); caudal lobe–β-endorphin-189 pmol · lobe^−1^, ACTH-383 pmol · lobe^−1^ (ratio 1:2.03) (Barna et al., 1998a).

### Physiological Roles of β-Endorphin

There is evidence that β-endorphin is involved in the inhibitory control of gonadotropin releasing hormone (GnRH) release from the hypothalamus at least in chickens. β-endorphin cell bodies in the periarcuate area project to the median eminence (Contijoch et al., 1993). Intra-ventricular administration of β-endorphin blocks the pre-ovulatory LH surge (Sakurai et al., 1986). Moreover, β-endorphin depresses *in vitro* GnRH release from hypothalamic tissue from hens during pre-ovulatory surge of luteinizing hormone (LH) (Contijoch et al., 1993)

## Met-Enkephalin in Birds

The Met-enkephalin together with Leu-enkephalin are pentapeptide endogenous opioids:

- Met-enkephalin - Tyr-Gly- Gly-Phe-Met (YGGFM)
- Leu-enkephalin - Tyr-Gly-Gly-Phe-Leu (YGGFL)

These neuropeptides were initially isolated from the brain of pigs (Hughes et al., 1975). Met- enkephalin and Leu-enkephalin are produced by proteolytic cleavage of pre-proenkephalin at paired-basic amino acid residues. Proenkephalin contains seven enkephalin motifs:

- Motifs 1-3 Met-E (Met-enkephalin)
- Motif 4 - MEAGL – octapeptide – Met-Enkephalin-Arg-Gly-Leu,
- Motif 5 - Met-E (Met-enkephalin),
- Motif 6 - Leu-E (Leu-enkephalin)
- Motif 7 MEAP - heptapeptide - Met-Enkephalin-Arg-Phe.

### Evolutionary Aspects of Enkephalins

Met- and Leu-enkephalin are identical across the vertebrates (Roberts et al., 2007). There are seven enkephalin motifs in proenkephalin (Figure 1) in chickens and across the gnathostomes (jawed vertebrates). While, Met-enkephalin is identical, there are differences in both the heptapeptide (e.g., amphibian, newt - YGGFMRY and zebrafish – YGGFMGY) and octapeptide (e.g.,YGGFMRGY in the primitive mammal-platypus and amphibians; YGGFMRSV – chickens) (Bojnik et al., 2010, 2011).

The PENK gene has not been identified in invertebrates. However, invertebrate tissues have been reported to contain proteins homologous to proenkephalin (Stefano and Salzet, 1999). Immuno-reactive Met-enkephalin has been detected in, for instance, crustaceans (Jaros et al., 1985), annelids (Tasiemski et al., 2000) and tunicates (Georges and Dubois, 1984). In addition, Leu-enkephalin and delta opioid receptor immuno-reactivity are found in a mollusk, the octopus (Sha et al., 2012). Moreover, enkephalins influence the physiology of invertebrates, for instance, disrupting reproduction in insects (Kumar et al., 2013) and impacting both heart and immune functioning in molluscs (Tasiemski et al., 2000; Liu et al., 2016).

### Circulating Concentrations of Met-Enkephalin

Plasma concentrations of Met-enkephalin in chickens are 28 pg · ml^−1^ (~50 fmoles · ml^−1^) (Pierzchala and Van Loon, 1990). There is a larger form of Met-enkephalin called cryptic ([Met^5^]–enkephalin) that found both in the circulation and in tissues; this being enzymatically cleaved (processed) to the pentapeptide (Pierzchala and Van Loon, 1990). Cryptic Met-enkephalin appears to be not only the storage of Met-enkephalin (free pentapeptide) but is also quickly processed under different stressors such as withholding of food and water, overcrowding, cold and restraint (Pierzchala-Koziec et al., 1999). Cryptic Met-enkephalin concentration in blood plasma of growing chickens was ~4.2 pmoles · ml^−1^ (Pierzchała-Koziec and Mazurkiewicz-Karasińska, 1997; Pierzchala-Koziec et al., Submitted). Enkephalins have short half-lives in the circulation. For instance, Leu-enkephalin is rapidly degraded by aminopeptidase M in chicken plasma with a half-life of 0.7–1.0 min *in vitro* (Shibanoki et al., 1991). Thus, aminopeptidases inhibitors need to be present in tubes and syringes when sampling birds.

Some stressors have been demonstrated to increase plasma concentrations of Met enkephalin in chickens. This is in addition to the stress induced activation of the hypothalamo-pituitary-adrenocortical (HPA) axis. Plasma concentrations of Met-enkephalin were increased by short term crowding in chickens with the effect being attenuated in the presence of the opioid antagonist, naltrexone (Pierzchała-Koziec et al., 1996; Pierzchala-Koziec et al., Submitted). Fasting increased plasma concentrations of Met enkephalin in female chickens but decreased those in male chickens (Pierzchała-Koziec and Mazurkiewicz-Karasińska, 1997; Pierzchala-Koziec et al., Submitted). Moreover, acute withholding of water was accompanied by increased plasma concentrations of Met-enkephalin (Pierzchala-Koziec et al., Submitted). What are not known are the effects of stress on Met-enkephalin in wild birds or the physiological relevance of increased plasma concentrations of Met-enkephalin during stress.

### Control of Met-Enkephalin Release

There is very limited information on control of Met-enkephalin release. In chickens, release of Met-enkephalin from the hypothalamus and pituitary gland *in vitro* is depressed in tissues taken from birds subjected to water deprivation stress (Mazurkiewicz-Karasińska and Pierzchała-Koziec, 1997; Pierzchala-Koziec et al., Submitted). There are increases in release of Met-enkephalin and cryptic [Met^5^]- enkephalin from the retina during the night/darkness in chickens (Dowton et al., 1990).

There is evidence that endogenous opioids depress *in vitro* release of Met-enkephalin from the hypothalamus. *In vitro* release of Met-enkephalin is increased when chicken hypothalamic fragments are incubated in the presence of naltrexone (Mazurkiewicz-Karasińska and Pierzchała-Koziec, 1997; Pierzchala-Koziec et al., Submitted). There are changes in release of Met-enkephalin and cryptic [Met^5^]- enkephalin from the retina during the night/darkness in chickens (Dowton et al., 1990).

There is some evidence of cross talk between the HPA axis and Met-enkephalin in birds. This is supported by the report that stress increases plasma concentrations of both Met-enkephalin, and corticosterone in chickens (Pierzchala-Koziec et al., Submitted). In addition, corticotropin releasing hormone (CRH) increases release of both Met-enkephalin (Pierzchala-Koziec et al., Submitted) and ACTH in chickens (Nakayama et al., 2011). Moreover, there is evidence that glucocorticoids influence release of Met-enkephalin with dexamethasone increasing release of Met-enkephalin *in vitro* from either chicken hypothalamic or adrenal tissue (Pierzchala-Koziec et al., Submitted).

### Distribution of Met-Enkephalin and Tissue Concentrations

Table 1 summarizes the available information on the distribution of Met-enkephalin. The distribution of enkephalin neurons has been reported in the pigeon brain (Bayon et al., 1980). Enkephalin neurons are found, for instance, in the brainstem, limbic regions, organum vasculosum hypothalamic, paleostriatum, and pituitary stalk (Bayon et al., 1980). There are marked differences in the distribution of enkephalin neurons and those containing β-endorphin (Bayon et al., 1980).

**Table 1:** Cells containing immuno-reactive Met-enkephalin in chicken tissues.

**Organ**	**Any details**	**References**
Adrenal gland	Both norepinephrine and epinephrine producing chromaffin cells	Ohmori et al., 1997
Gastro-intestinal tract	Gizzard- myenteric plexus and the outer circular muscle	Jiménez et al., 1993
	But not in proventriculus	Martínez et al., 2000
	Small intestine: myenteric and the deep muscular plexuses	Jiménez et al., 1993
	Ceca: neurones projecting into the ceca	Ohmori et al., 2003
Posterior pituitary gland	Mesotocin containing neurosecretory terminals	Martin et al., 1992
Retina		Dowton et al., 1990
Spinal cord	Nerve fiber- and terminal-like processes of the lumbar spinal cord	Maderdrut et al., 1986
Thymus	Endocrine cells	Atoji et al., 1997

Tissue concentration of Met-enkephalin and PENK gene expression have been determined.

- Met-enkephalin concentrations in chickens: Pituitary > hypothalamus> adrenal gland > cerebellum >heart>kidney (Pierzchala-Koziec et al., Submitted).
- PENK expression: Pituitary > hypothalamus > adrenal gland > cerebellum >> heart atria > heart ventricles > kidneys (Pierzchala-Koziec et al., Submitted).

Tissue concentrations of Met-enkephalin change during avian embryonic development. There are large increases (>1,000 fold) in immuno-reactive (IR-) Met-enkephalin in the lumbar spinal cord between days 4.5 and 18 in chick of embryos (Maderdrut et al., 1986). Moreover, IR-Met-enkephalin is not observed in the circular smooth muscle until day 17 of embryonic development (Epstein et al., 1983).

### Physiological Role of Met-Enkephalin

Met-enkephalin may act in neuromodulatory, and/or hormonal and/or paracrine manners. Met-enkephalin inhibits release of gonadotropin releasing hormone (GnRH) from hypothalamic tissue from male chickens *in vitro* with the effect being via mu opioid receptors (Stansfield and Cunningham, 1987, 1988). It is unclear whether these observations reflect an effect of Met-enkephalin *per se* or exogenous Met-enkephalin acting as a surrogate for other endogenous opioids. Met-enkephalin has been reported to have other effects in birds. For instance, Met-enkephalin acts as a gastric inhibitor in chickens via mu receptors (Jiménez et al., 1993).

## Dynorphin in Birds

There is very limited information on dynorphin in birds. The structures of chicken dynorphins can be deduced from the cDNA:

- Dynorphin A - YGGFMRRIRPKLKWDN
- Dynorphin B - YGGFLRRQFKVTT

(based on Figure 1). An additional peptide product is α-neo-endorphin.

### Circulating Concentrations of Products of the Prodynorphin

There are no reports on circulating concentrations of dynorphin A or B in birds. However, overcrowding stress was associated with increased plasma concentrations of α-neo-endorphin (Pierzchała-Koziec et al., 1996).

### Distribution of Dynorphin

The distribution of dynorphin neurons in the pigeon brain has been reported (Reiner, 1986). Outside of the hypothalamus, there are neurons containing both substance P and dynorphin (Reiner, 1986).

### Physiological Effects of Dynorphin

There is evidence that dynorphin plays a role in the neuroendocrine control of prolactin release in birds. Intraventricular infusion of dynorphin into the II ventricle is followed by increases in circulating concentrations of prolactin in turkeys with the effect mediated via kappa (κ) opioid receptors as demonstrated by κ opioid receptor antagonists to block the effect (Youngren et al., 1993, 1999).

## Other Neuroendocrine Effects of Endogenous Opioids in Birds

There is evidence that endogenous opioids influence release of arginine vasotocin (AVT) in birds. Plasma concentrations of AVT, but not those of mesotocin, are increased by morphine and a specific mu receptor agonist in chickens (Saito et al., 1999; Sasaki et al., 2000). Moreover, AVT release is depressed by the opioid receptor antagonist, naloxone, in hypertonic saline treated chickens (Saito et al., 1999). It is not clear which endogenous opioid influences AVT release. It is reasonable to exclude dynorphin as it is not present in the pars nervosa of the chicken (Martin et al., 1992). Opioid agonists appear to inhibit whereas antagonists stimulate socio-sexual interactions in starlings (Riters, 2011). Moreover, it was suggested that enkephalin in medial preoptic area is involved in reward associated with both feeding and sexual behavior (Riters, 2011).

## Conclusions and Future Directions

There are few studies of endogenous opioids in poultry species and none in wild birds. The available evidence that both β-endorphin and Met-enkephalin are present across the class *Aves*. The structure of β-endorphin is very similar across birds suggesting its importance. There have been some physiological studies and all support a relationship between both β-endorphin and Met-enkephalin and stress. What is missing are comprehensive studies of plasma concentrations, expression and receptors of the full array of endogenous opioids in multiple avian species under the following situations:

- Temporal changes during annual, daily/circadian and ovulatory cycles.
- Acute responses in response to activators of the HPA axis, other stressors (e.g., disease), neuropeptides and hormones.
- Interactions between the different endogenous opioids and their receptors.

There is considerable scope for investigation of the physiology of endogenous opioids in birds.

## Author Contributions

KP-K and CS: conceived, drafted and wrote the review. It is based in part on research from KP-K's laboratory. CS and KP-K: re-analyzed the research and together discussed their relevance.

### Conflict of Interest Statement

The authors declare that the research was conducted in the absence of any commercial or financial relationships that could be construed as a potential conflict of interest.
